# Supplementary material for: Chromothripsis during telomere crisis is independent of NHEJ, and consistent with a replicative origin
Source: Genome Res. 2019 May;29(5):737–49. doi: 10.1101/gr.240705.118 (PMC6499312; doi:10.1101/gr.240705.118)
Supplement: Supplemental Material [file supp_gr.240705.118_Supplemental_file_1.zip › contigs/annotated_contigs/DB57/contig.2.DB57_length_275_mean_cov_16.0981818182.docx]

**DB57_length_275_mean_cov_16.0981818182**

```
GGGGGGGGGGGGGGGGGGGGGGGGGGGGGGGGGGGGGGGGGGGAGGGGGGGGGGGAGGAGGGGAG|GGGG|AGGGAGAGAGAGGGGGAG
 >chr2:33141565-33141634 + E=3e-21 p=0e+00                        >chr22:34792172-34792
GGGGAGAGAGAGAGAGAGAGATTGAGATTGGAGAAAATGGGTTAGATTGATTTGGCCAACATATGTGCATATTTTTTATACAAGCCTCT
380 - E=3e-100
TGGATTTAGTCTTATCTGTTGATCCATGCTCCAAGGCTAGGGAAACAGCATCCCTGCTTTTGTCTGTATCTTAATTAATTCATTGATTC

TGTTGCGATA
```
